# Quality Assessment of the Genetic Test for Familial Hypercholesterolemia in The Netherlands

**DOI:** 10.1155/2013/531658

**Published:** 2013-07-08

**Authors:** Iris Kindt, Roeland Huijgen, Marieke Boekel, Kristiaan J. van der Gaag, Joep C. Defesche, John J. P. Kastelein, Peter de Knijff

**Affiliations:** ^1^Foundation for the Identification of Persons with Inherited Hypercholesterolemia (StOEH), Amsterdam, The Netherlands; ^2^Department of Vascular Medicine, Academic Medical Center (AMC), 1105 AZ Amsterdam, The Netherlands; ^3^Department of Human Genetics, Forensic Laboratory for DNA Research, Leiden, The Netherlands

## Abstract

*Introduction*. Familial hypercholesterolemia (FH) is an inherited disorder associated with a severely increased risk of cardiovascular disease. Although DNA test results in FH are associated with important medical and ethical consequences, data on accuracy of genetic tests is scarce. *Methods*. Therefore, we performed a prospective study to assess the overall accuracy of the DNA test used in the genetic cascade screening program for FH in The Netherlands. Individuals aged 18 years and older tested for one of the 5 most prevalent FH mutations, were included consecutively. DNA samples were analyzed by the reference and a counter-expertise laboratory following a standardized procedure. *Results*. 1003 cases were included. In the end, 317 (32%) carried an FH mutation, whereas in 686 (69%) samples no mutation was found. The overall accuracy of the reference laboratory was 99.8%, with two false positive results identified by the counter-expertise laboratory. *Conclusion*. The currently used mutation analysis is associated with a very low error rate. Therefore, we do not recommend routine use of duplicate testing.

## 1. Introduction

The number of hereditary disorders for which a genetic test is available has increased from less than 200 in 1993 to more than 1,800 in 2009 [1]. Molecular genetic testing for these diseases raises a plethora of concerns, including the quality of test performance and interpretation of results [2].

Familial hypercholesterolemia (FH) is a condition that meets every criterion for genetic screening. FH is an autosomal codominant disorder of lipid metabolism with a prevalence of 1 : 500 in most Western countries [3]. Patients with FH have high plasma LDL cholesterol levels and an increased risk of coronary artery disease (CAD) [3, 4]. Statin therapy, intervening in the causal pathway of the disease, lowers CAD risk to a very significant extent in these individuals [5]. Defects in genes that code for proteins involved in hepatic clearance of low-density lipoprotein (LDL) cholesterol underlie the disorder [3]. In fact, more than a 1000 different mutations in the genes coding for the LDL-receptor (*LDLR*), apolipoprotein B (*APOB*), and proprotein convertase subtilisin/kexin type 9 (*PCSK9*) are known to cause FH [6]. Such a causal mutation can be identified by DNA analysis in a varying percentage of patients with a clinical FH diagnosis, ranging from 20% to more than 90%, with highest rates of detection in children strictly selected for severe clinical FH [7–13]. Knowledge of the causal monogenetic mutation enables rapid screening of family members for the presence of the same mutation. In fact, such a genetic cascade screening programme for FH started in The Netherlands in 1994 and was scaled up in 2003 with the aid of government support [14].

On an individual level, FH tests can have important medical and social/ethical consequences. The vast majority of individuals initiate cholesterol-lowering treatment after such a diagnosis [15]. Conversely, a person's test result may affect employment or the ability to secure life insurance [16, 17]. Because the results of genetic testing can have such a profound impact on the life of screened individuals, a high standard is required for the accuracy of DNA analysis. However, the reproducibility of DNA testing for FH has not been evaluated and hitherto no gold standard exists for DNA-based diagnostic testing for this condition. 

Therefore, we decided to assess the quality of the DNA test results in our screening programme. To arrive at this, we conducted a prospective study in which two independent laboratories tested samples in duplicate using a systematic procedure. Here, we present our results.

## 2. Methods and Materials

### 2.1. Selection and Recruitment of Patients

In a prospective study, we aimed to include 1000 consecutive subjects that were tested for genetic FH. These subjects were recruited from the participants of the cascade screening program for FH in The Netherlands from November 2007 until December 2009. We only selected individuals aged 18 and older and those that were to be tested for one of the five most prevalent FH mutations. In general, the carriers of these five mutations represent approximately 50% of the molecularly diagnosed FH patients [18, 19].

The selected subjects were asked for consent for the genetic screening for FH and, in addition, for this study, that is, assessment of the reproducibility of the FH mutation analysis. The genetic cascade screening for FH and this sub study was approved and financed by the Dutch Government (RIVM). 

After written informed consent was obtained, trained nurses from the StOEH drew blood from each participant. From heparinized blood the lipid profiles were measured with the LDX analyser [20]. The LDL cholesterol was estimated based on the Friedewald formula [21]. Age and sex specific percentiles of LDL cholesterol were calculated using the reference values of the Caucasian population [22]. 

Four vacutainers (DB Vacultainer, 5.0 mL) containing EDTA as anticoagulant were drawn for DNA extraction for mutation analysis.

### 2.2. Mutation Analysis

Two samples were sent to and processed by the laboratory for Experimental Vascular Medicine of the Academic Medical Center at the University of Amsterdam (referred to as the reference laboratory). The other two vacutainers were sent to and processed by the counter-expertise laboratory of the National Forensic Institute laboratory in Leiden (referred to as the counter-expertise laboratory). The counter-expertise laboratory performed its tests after the reference laboratory and was blinded for the findings of the reference laboratory. 

Four mutations in *LDLR* and one in *APOB *were tested in this study (See Supplemental Table  1 (main characteristics of mutations) in Supplementary Material available online at http://dx.doi.org/10.1155/2013/531658). Mutations were described according to the nomenclature as proposed by den Dunnen and Antonarakis [23].

### 2.3. DNA Analysis in the Reference Laboratory

Genomic DNA was isolated from the tubes with 5 mL whole blood on an AutopureLS apparatus according to a protocol provided by the manufacturer (Gentra Systems, Minneapolis, USA). The remainder of the blood was stored at −20°C. Mutations were detected by amplification of the exon harbouring the mutation in question by polymerase chain reaction (PCR), followed by digestion of the PCR products with an appropriate restriction endonuclease and gel electrophoresis on agarose gels to separate the digestion products. The primer sequences and conditions for PCR are available upon request. The presence or absence of a mutation was determined by the difference in digestion pattern, as described previously [24, 25].

### 2.4. DNA Analysis in the Counter-Expertise Laboratory

From each tube 200 *μ*L blood was used for DNA extraction using the Qiacube DNA isolation robot (Qiagen Germany) using standard protocols provided by the manufacturer. The remainder of the blood was stored at −20°C. DNA typing was performed using a multiplex SNaPshot assay designed to detect the specific mutations studied in this study. Reference sequences for each mutation of the *LDLR* and *APOB* were obtained from the literature [6, 26, 27]. The primers that were designed are available upon request. Specifics of DNA typing reactions are described in Supplemental File 1. Data was analyzed using GeneMapper ID v3.2.1 (Applied). After background subtraction and colour separation, peaks were sorted into bins according to sizes by comparison to the internal size standard. An Excel-sheet was used to transfer exported allele tables and automatically call mutations.

The overall costs of setting up the logistics for the DNA typing of the 5 different mutations, including personnel and materials, were €150 per individual tested.

### 2.5. Combining the DNA Test Results of Both Laboratories

The test results of the two laboratories were reported to the StOEH, and two individuals (MB RH) independently reviewed the test results for discrepancies. Samples with discrepant results between the two laboratories were analysed according to the subsequent steps until the cause of the discrepancy was discovered. If required, we used the following steps.

- Step 2: re-analysis of the same DNA sample as in the first analysis (performed at both labs);
- Step 3: new DNA extraction and analysis from the second tube of the sample (both labs);
- Step 4: re-analysis of the second DNA sample (both labs);
- Step 5: exchange of DNA sample from the first DNA extraction between the reference and counter-expertise lab and re-analysis of this DNA sample;
- Step 6: new sampling of blood from the subject in question and initiation of Step  1 and subsequent steps in both labs.

### 2.6. Outcomes

In case of discrepant results between the laboratories, the end conclusion of subsequent steps was that the initial conclusion of the reference laboratory was erroneous or not, and, if an error was discovered, whether the initial mutation analysis result was false positive or negative. The main study outcome was to answer whether the current DNA analysis for genetic FH by the reference laboratory provided test results that were compatible in at least 99.5% of cases with the gold standard. The gold standard was the presence or absence of the mutation based on the overall conclusion of stepwise analysis of the counter-expertise laboratory and the reference laboratory. 

Secondary outcomes were (i) the stage at which an error had been made during meta-analysis and (ii) the costs made by the counter-expertise laboratory to identify one erroneous result made by the reference laboratory and the added value of routinely typing of all 5 mutations in duplicate.

### 2.7. Statistical Analysis

#### 2.7.1. Sample Size Calculation

Assuming a discrepancy percentage of 0.5% between the results of the two laboratories with a confidence interval of 2% and using a 2-sided alpha level of 0.05 at 80% power, 1000 patients would be needed.

We compared differences in lipid levels between carriers and noncarriers with an independent *t*-test. All data were analyzed using SPSS software (version 16.0.2, SPSS, Chicago, MI, USA). A *P* value of less than 0.05 was considered to be statistically significant. 

## 3. Results

In total, 1003 participants were included.  Table 1 summarizes the main characteristics of the participants and the mutations tested. The participants had a mean age of 49 years, and 520 (52%) were males. The two mutations that were analyzed most often were those that resulted in changes of the arginine at position 3527 in *APOB* (*N* = 348) and the p.N564H/2393del9 bp in *LDLR* (*N* = 317), as could be expected based on the prevalence of these mutations. The other three mutations were tested in 330 individuals. In addition, eight individuals had been tested for a special mutation, which consisted of the c.191−2 and the c.313+1 mutations residing on the same allele of the *LDLR*. 

**Figure 1:**
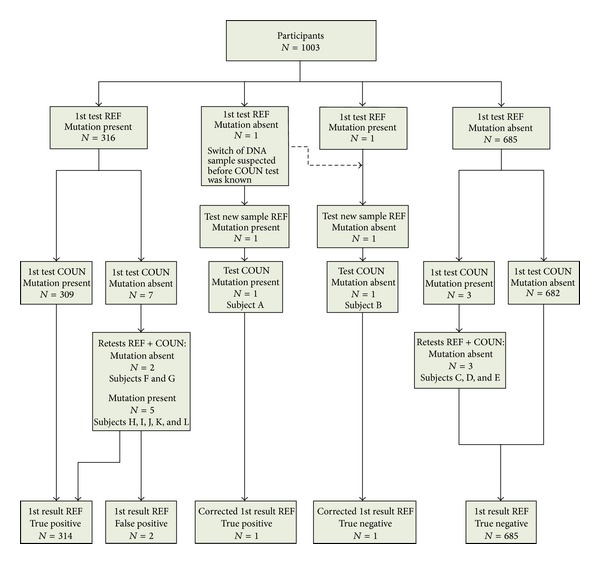
Patient enrolment and flow diagram based on mutation analysis. Diagnostic steps used for the participants. “True positive”, “False positive,” and “True negative” were based on end conclusion on mutation carriership after stepwise analyses in both laboratories, which indicate whether the laboratory result from the reference laboratory proved correct (True positive or True negative), or incorrect (False positive). Abbreviation: COUN = counter-expertise laboratory, REF = reference laboratory.

Mutation analysis of the 1003 DNA samples in the reference laboratory revealed the presence of a mutation in 317 (32%) samples, whereas in the other 686 (69%) samples the mutation proved absent.  Figure 1 illustrates the diagnostic procedure followed for the participants.  Table 2 summarizes the overall test results and clinical characteristics of specific persons of interest. In case of subject A (Figure 1 and Table 2), the trained nurse suspected that the conclusion by the reference laboratory was incorrect, even before the counter-expertise laboratory had initiated the analysis. This case involved a 43-year-old male who, according to the initial findings of the reference laboratory, did not carry the p.N564H/2393del9 bp mutation. He had experienced a myocardial infarction at the age of 41 years. Because his medical history and his lipid profile were indicative of FH (Table 2), the cascade screening organization requested that the reference laboratory reanalyzed this individual. When retrieving the different steps of the analytical procedure it became clear that the blood sample of individual A was exchanged for that of individual B, who was analyzed for the same mutation (Table 2). Re-analysis of the DNA extracted from the spare tube of A and B revealed the presence of p.N564H/2393del9 bp variant in individual A and the absence of it in individual B. These findings were confirmed by the counter-expertise laboratory (Figure 1). 

Mutation analysis of the other 1001 samples resulted in 10 discrepant results between the counter-expertise and reference laboratories. Supplemental Table 2 illustrates the end conclusion on the test results which initially gave discrepant results between both laboratories. In three cases, the counter-expertise laboratory identified a mutation, whereas the reference laboratory did not (cases C, D, and E, Figure 1). In these cases the LDL-cholesterol levels were below the 40th percentile for age and gender, which made the presence of FH less credible (Table 2). Based on the subsequent re-analyses by both laboratories, we concluded that those three individuals did indeed not carry a mutation. An error had occurred in the counter-expertise laboratory, where the samples of these subjects were exchanged for three others that carried a mutation (Supplemental Table 2). Thus, the initial findings of the reference laboratory were correct. 

The counter-expertise laboratory identified absence of the mutation in seven samples in their first test, where the reference laboratory initially had found a mutation (Figure 1). In individuals F and G, subsequent analyses in both labs revealed that the first test result from the reference laboratory was indeed false positive (Figure 1). Both individuals had not experienced cardiovascular disease and had normal cholesterol levels, with LDL-cholesterol levels below the 40th percentile for age and gender (Table 2). Individual F was repeatedly tested homozygous positive for the 313+1G>C mutation in the first analysis in the reference laboratory, while in tests on the spare sample the mutation was absent. Ultimately, sequence analysis of the first and the spare sample confirmed the absence of this mutation. The reason for a repeated homozygous but false positive test result in PCR analysis remains unknown, but maybe related to the quality of the DNA sample (Supplemental Table 2). Sample switch during DNA extraction was excluded because re-analysis of the spare samples, which were processed in the same batch, did not yield conflicting results with the contra-expertise laboratory. The false positive result in individual G was shown to be caused by a sample switch between the consecutive sample of an individual that was analyzed for the same mutation (Supplemental Table 2).

The five other discrepancies involved individuals H, I, J, K, and L, in whom the initial test of the counter-expertise laboratory had identified absence of the mutation. The clinical characteristics of individuals H, I, and J were indicative of presence of an FH mutation, while clinical data of K and L were incomplete (Table 2). Indeed, the end conclusion after stepwise analyses was that the FH mutation was present in these five individuals, and the findings of the reference laboratory were correct. In cases H and I the counter-expertise laboratory failed to identify the mutation during the first test, because the diagnostics were initially only designed to detect the common nucleotide changes at that position. Cases H and I, should have been tested for the mutations c.10579C>T (or p.R3527W) in *APOB* and 313+1G>C in *LDLR*, respectively. However, the counter-expertise laboratory was initially only equipped to detect these common variants at those positions, which were c.10580G>A for p.R3527Q in *APOB* and c.313+1G>A in *LDLR*, respectively (Supplemental Table  1). The mismatch was solved after counter check laboratory expanded the diagnostic procedure to also detect those rare variants. The mismatches in cases J, K, and L were due to the exchange of the samples with those of noncarriers C, D, and E by the counter-expertise laboratory, as described before (Supplemental Table 2).

Supplemental Table 3 shows the mean lipid levels and proportion of medication use for carriers and noncarriers categorized for the different mutations. This table illustrates the extent of dyslipidemia observed in the carriers of one of those specific mutations.

Overall, two false positive test results were discovered by the counter-expertise laboratory (subjects F+G). As a consequence, the sensitivity of the first DNA test by the reference laboratory was 1.0, and the specificity (95% CI) was 0.997 (0.891 to 1.00) (Table 3). Conversely, eight incorrect conclusions of the first tests of the counter-expertise laboratory were discovered during this study. Two of those were caused by a mistake in setting up the replication of the mutations, where the rare variants were initially “not covered.” The other errors were due to three erroneous exchanges of samples in the pre-analytic phase. 

## 4. Additional Findings by the Counter-Expertise Laboratory

The counter-expertise laboratory used a mutation analysis test where all 5 different mutations were tested simultaneously. As a consequence of this procedure, the counter-expertise laboratory identified an unexpected mutation—so another mutation than the one requested to analyse in duplicate—in two subjects. 

In one 32-year-old male (Individual M, Table 2), the requested mutation was the *LDLR*-c.1359−1, which was absent according to the analyses of both labs. However, the counter-expertise laboratory did identify the *LDLR*-c.313+1G>A mutation. The patient had been identified with elevated total cholesterol by the general physician before the study visit and used rosuvastatin 40 mg. Despite this treatment, the patient still had a rather unfavourable lipid profile. Thus, the presence of the *LDLR*-c.313+1G>A mutation fully accounts for the clinical FH phenotype (Table 2), whereas the first test result of the reference laboratory, that is, absence of the initial FH mutation *LDLR*-c.1359−1, would have left the phenotype with hypercholesterolemia unexplained. 

Similarly, both labs excluded the presence of the *LDLR*-c.313+1G>A mutation in a 62-year-old man from another family (no complete lipid profile determined because of high triglycerides), but the counter-expertise laboratory identified an *APOB*-p.R3527Q mutation. Subsequently, his direct relatives were tested for the presence of this *APOB* mutation as well, and this led to the identification of a relative that carried both *LDLR*-c.313+1G>A and the *APOB*-p.R3527Q mutation (Individual N, Table 2). This involved a 68-year-old male, whose total cholesterol level was 17.5 mmol/L before he had initiated treatment.

## 5. Discussion

In our study, a duplicate DNA analysis for the molecular diagnosis of FH was performed in 1003 relatives of patients with an established DNA diagnosis of FH. The findings suggest that the DNA mutation analysis, currently being used by the cascade screening for FH (including a second analysis in case of conflicting clinical and mutation results), is associated with a low error rate. In fact, the counter-expertise laboratory discovered no more than two false positive cases (0.2%) from the national reference laboratory. 

Implementing a duplicate test for the 1003 individuals came at a cost of approximately €150,000. This would constitute an increase of 37%, compared to the original cost per visited family member, including DNA analysis. Thus, the price tag was €150,000 for the discovery of two erroneous test results from the reference laboratory. In particular, this price was based on the costs associated with setting up an assay and duplicate testing of the five most prevalent mutations in The Netherlands. The cost efficiency of implementing a duplicate DNA test will likely be much lower if specific duplicate tests would have to be set up for all the mutations that were found to cause FH in The Netherlands. Of note, more than 400 pathogenic FH mutations have now been identified in The Netherlands, and most of these are rare [18, 28]. In general, it is highly desirable to eliminate erroneous results of DNA tests. We argue, however, that the overall accuracy of 1001 out of 1003 (99.8%) of the initial DNA test is within acceptable limits. The efficiency of the duplicate DNA test is too little, in our opinion, to routinely implement such a procedure.

Even so, the study findings can assist us in improving the logistics for mutation analysis. The erroneous test results were primarily due to switching of blood or DNA samples in the pre-analytic phase. This emphasizes that blood handling during DNA extraction and preparation of the DNA for mutation analysis should always be performed at utmost accuracy and guarded by standard operating procedures.

Furthermore, our findings show that good clinical judgement and critical evaluation of mutation test results remain essential to conclude on genetic FH status. A good example of this is individual A (Figure 1 and Table 2). The initial genetic test did not reveal presence of the mutation, which was surprising, because his clinical characteristics suggested that he did have FH. This triggered a trained nurse to request a re-analysis. Based on that process a switch of blood samples was discovered before the counter-expertise laboratory confirmed the mistake. 

If systematic follow up would have been performed in those cases where genetic test results did not fit the clinical phenotype, several other mutation analysis errors could have been detected. In fact, the two false positive test results from the reference laboratory were not supported by a clinical FH phenotype.

Additional findings were (1) most of the false negative and false positive tests from the counter-expertise laboratory could have been discovered based on a mismatch between mutation analysis result and the clinical phenotype (Table 2 and Supplemental Table  3) (2) the discovery of 2 mutations which were not found in the initial analysis. In our study cohort we observed this phenomenon twice: the initial mutation known to cause FH in the family was absent, but another FH mutation was picked up by the counter-expertise laboratory (see Individuals M and N from  Table 2). (3) Lastly, in case a person with a genetic diagnosis of heterozygous FH has exceptionally severe dyslipidemia, homozygous FH or compound heterozygosity should be considered [29]. This is what we observed for individual N, who had pre-treatment total cholesterol level of 17.5 mmol/L and who was shown to carry pathogenic mutationsin both* LDLR* and *APOB *genes. 

Our study design had several limitations. First, a proper gold reference for DNA testing does not exist. In absence of a gold standard, we used a composite measure where, if applicable, several steps of the standard DNA-test and a comparator DNA test would lead to a best estimate. 

Second, errors in the pre-analytic phase might have remained unnoticed, for example, if blood samples were accidentally switched between family members visited simultaneously before these were send to the two labs. Therefore, our results should primarily be used to get a sound impression on the processes of extraction of DNA, typing and interpretation of the results.

Third, our study results are only applicable to a subset of families with FH with clear monogenetic FH. An elegant analysis performed in large clinical FH population in the United Kingdom and Belgium showed that in a large proportion of patients no causal monogenic FH mutation can be identified [30]. In the mutation negative clinical FH patients more than half had a high allele count of common LDL cholesterol-raising variants, suggesting a polygenic cause of their hypercholesterolemia. By design, our analysis only addressed the FH families with a clear large effect monogenetic mutation in *LDLR* and *APOB *genes, where cascade screening of that variant was performed. 

Last, our analysis was solely designed to assess the accuracy of the methodology used in the reference laboratory from the cascade screening programme. Because other professional laboratories might use SNP assays or direct sequencing instead, the results of the current study can not always be extrapolated for genetic testing programmes performed in other countries. 

In conclusion, we would argue that the currently used mutation analysis methodology is associated with an acceptable error rate and routine use of duplicate tests has a high price tag. Therefore, we do not recommend routine use of duplicate testing. 

## Supplementary Material

Supplemental table 1: Main characteristics of the mutations.
Supplemental table 2: Discrepant test results between the reference and counter-expertise laboratory.
Supplemental table 3: Mutation carriership medication use and lipid levels.
Supplemental file 1: DNA-typing in the counter-expertise laboratory.

## Figures and Tables

**Table 1:** Baseline characteristics of the participants.

	All
*N *	1003
Age (±SD) years	49 ± 16
Male gender (%)	523 (52%)
Body mass index (±SD) kg/m^2^	25.6 ± 3.9
Medication use at diagnosis (%)	250 (25%)
Tested for mutation (% of total)	
* LDLR*-c.313+1/2	140 (14%)
* LDLR*-p.S306L	80 (8%)
*LDLR*-c.1359−1G>A	110 (11%)
* LDLR*-p.N564H/2393del9bp	317 (32%)
* APOB*-p.R3527 L/Q/W	348 (35%)
* LDLR*-c.313+1/2+c.191−2	8 (1%)

Based on the per protocol study population: subjects tested when aged under 18 years were excluded.

**Table 2:** Clinical characteristics of participants of interest.

ID	Sex	Age	Medication use	TC (perc.)	HDL (perc.)	LDL (perc.)	TG (perc.)	Mutation tested	Initial finding^#^	End conclusion*
A	Male	43	Simva 40	6.33 (86)	0.59 (<5)	3.87 (67)	4.09 (>95)	p.N564H/2393del9bp	Absent > present	Carrier
B	Female	65	Simva 40	4.26 (<5)	0.80 (<5)	2.80 (<5)	3.02 (>95)	p.N564H/2393del9bp	Present > absent	Noncarrier
C	Male	54	No	5.92 (68)	1.55 (93)	3.36 (36)	2.20 (83)	p.N564H/2393del9bp	Absent	Noncarrier
D	Male	55	No	4.86 (24)	1.50 (82)	2.64 (10)	1.56 (62)	p.S306L	Absent	Noncarrier
E	Female	56	No	4.05 (<5)	2.22 (91)	1.61 (<5)	0.50 (<5)	p.N564H/2393del9bp	Absent	Noncarrier
F	Male	54	No	5.17 (37)	1.14 (51)	3.42 (39)	1.32 (41)	c.313+1G>C	Present	Noncarrier
G	Male	38	No	3.20 (<5)	1.33 (82)	1.22 (<5)	1.41 (57)	p.R3527W	Present	Noncarrier
H	Female	65	Rosuva 40 + Ezetimibe 10	3.09 (<5)	0.84 (<5)	1.49 (<5)	1.66 (69)	p.R3527W	Present	Carrier
I	Female	58	Simva 40	6.15 (61)	1.81 (70)	3.76 (51)	1.27 (50)	c.313+1G>C	Present	Carrier
J	Male	26	No	7.00 (>95)	1.71 (>95)	4.88 (>95)	0.89 (39)	p.S306L	Present	Carrier
K	Female	35	No	n.p.	n.p.	n.p.	n.p.	p.R3527Q	Present	Carrier
L	Male	18	No	n.p.	n.p.	n.p.	n.p.	p.R3527Q	Present	Carrier
M	Male	32	Rosuva 40	5.34 (69)	0.90 (16)	4.15 (88)	0.61 (9)	c.1359−1G>A	Absent	Noncarrier c.1359-1, carrier c.313+1G>A
N	Male	68	Atorva 80 + Ezetimibe 10	5.37 (44)	1.09 (33)	3.68 (45)	1.30 (53)	c.313+1G>A	Present	Carrier of both c.313+11G>A and p.R3527Q mutations

^#^Initial finding indicates the conclusion after the initial test by the reference laboratory only. *End conclusion on mutation carriership was defined by the end result of the gold standard test in this study, which was the conclusion based on results from analyses in the reference laboratory and the counter-expertise laboratory. Abbreviations: TC: total cholesterol, HDL: high-density lipoprotein cholesterol, LDL: low-density lipoprotein cholesterol, TG: triglycerides, Perc.: percentile for age and sex. Simva: simvastatin, Rosuva: rosuvastatin, n.p.: not performed (lipid profile not measured because subject was not in fasted state).

**Table 3:** Frequencies of mutation detection in the per-protocol study population.

Test result	Gold standard test^#^		Total
	Mutation present	Mutation absent	
Reference lab: mutation present	314	2	**316**
Reference lab: mutation absent	0	685	**685**
Total*	**314**	**687**	**1001**

*Excluded the 2 individuals in whom switch of DNA samples was discovered before counter-expertise test had been performed (subjects A and B from Figure 1 and Table 2).

^#^Gold standard test is defined as the overall conclusion after following the steps for mutation analysis in both the reference laboratory and the counter-expertise laboratory.

Overall accuracy = 999/1001 = 0.998, 95%. Confidence interval: 0.998 − (1.96∗sqr(0.998∗(1.0–0.998))) to 0.998 + (1.96∗sqr(0.998∗(1–0.998))) = 0.911 to 1.00.

Sensitivity = 314/314 = 1.0, 95%. Confidence interval: 1.0 − (1.96∗sqr(1.0∗(1.0–1.0))) to 1.0 + (1.96∗sqr(1.0∗(1–1.0))) = 1.00.

Specificity = 685/687 = 0.997, 95%. Confidence interval: 0.997 − (1.96∗sqr(0.997∗(1–0.997))) to 0.997 + (1.96∗sqr(0.997∗(1–0.997))) = 0.891 to 1.00.

Likelihood ratio for a positive test = sensitivity/(1 − specificity) = 344, indicating that a positive result is 344 times more likely to occur in someone with the mutation according to the gold standard than in one without it.
