# Supplementary material for: Quality Assessment of the Genetic Test for Familial Hypercholesterolemia in The Netherlands
Source: Cholesterol. 2013 Jul 8;2013:531658. doi: 10.1155/2013/531658 (PMC3722838; doi:10.1155/2013/531658)
Supplement: Supplementary file 1 — Supplemental table 1: Main characteristics of the mutations. Supplemental table 2: Discrepant test results between the reference and counter-expertise laboratory. Supplemental table 3: Mutation carriership medication use and lipid levels. Supplemental file 1: DNA-typing in the counter-expertise laboratory. [file 531658.f1.doc]

Supplemental Table 1

| **Gene** | **Location** | **Nucleotide change** | **New name^a^** | **Old name^b^** |
| --- | --- | --- | --- | --- |
| *LDLR* | Intron 3 | c.313+1G>A* |  |  |
| *LDLR* | Intron 3 | c.313+1G>C* | c.313+1/2* | 313+1/2* |
| *LDLR* | Intron 3 | c.313+2T>C* |  |  |
| *LDLR* | Exon 6 | c.917C>T | p.S306L | S285L |
| *LDLR* | Intron 9 | c.1359-1G>A | c.1359-1G>A | 1359-1 |
| *LDLR* | Exon 11+17 | c.[1690A>C;2393_2401del] | p.N564H/2393del9bp | N543H/2393del9bp |
| *APOB* | Exon 26 | c.10580G>A | p.R3527Q | R3500Q |
| *APOB* | Exon 26 | c.10579C>T | p.R3527W | R3500W |
| *APOB* | Exon 26 | c.10580G>T | p.R3527L | R3500L |

The following reference sequences were used: LDLR: NM_000527.3 and APOB: NM_000384.2. Numbering of the nucleotides of the *LDLR* and *APOB* genes was based on the cDNA, with +1 being the A of the ATG translation initiation codon.

^a^New name represents numbering of the codons with the initiation codon is 1.

^b^Old name represents numbering of the codons with initiation codon is -21 of the *LDLR* and -27 for *APOB*

***Because a single PCR detects three variants (c.313+1 G>A, c.313+1G>C, c.313+2T>C) without discriminating between those, these three mutations are depicted as one type of mutation, although the c.313+1G>A is a frequent variant whereas the other two variants are rare.

**Supplemental table 2**: Discrepant test results between the reference and counter-expertise laboratory.

| **ID** | **Mutation tested** | **First test REF** | **Final test REF** | **First test Counter-expertise (COUN)** | **Final test COUN** | **Conclusion*** | **Cause mismatch REF and COUN first test result** |
| --- | --- | --- | --- | --- | --- | --- | --- |
| C | p.N564H/2393del9bp | Absent | Absent | Absent, but presence p.R3527Q | Absent | Non-carrier | COUN: Switch of sample of C with K or L in the pre-analytic phase |
| D | p.S306L | Absent | Absent | Absent, but presence p.R3527Q | Absent | Non-carrier | COUN: Switch of sample of D with K or L in the pre-analytic phase |
| E | p.N564H/2393del9bp | Absent | Absent | Absent, but presence p.S306L | Absent | Non-carrier | COUN: Switch of sample of E with J in the pre-analytic phase |
| F | c.313+1 G>C | Present | Absent | Absent | Absent | Non-carrier | REF: Initial PCR-restriction enzyme reaction yielded erroneous false positive results whereas repeats with PCR-restriction as well as sequencing on DNA extracted from the same blood sample and from spare blood sample yielded wildtype results |
| G | p.R3527W | Present | Absent | Absent | Absent | Non-carrier | REF: Switch of sample of G in the pre-analytic phase with that of a consecutive patient tested for that mutation (that patient did not participate in this reproducibility study). |
| H | p.R3527W | Present | Present | Absent^#^ | Present | Carrier | COUN: lab had initially designed test only to detect the p.R3527Q mutation and not p.R3527W. This was corrected |
| I | c.313+1 G>C | Present | Present | Absent^#^ | Present | Carrier | COUN: lab had initially designed test only to detect the c.313+1G>A and c.313+2T>C mutation and not the rare c.313+1G>C: This was corrected |
| J | p.S306L | Present | Present | Absent | Present | Carrier | COUN: Switch of sample of J with E in the pre-analytic phase |
| K | p.R3527Q | Present | Present | Absent | Present | Carrier | COUN: Switch of sample of K with C or D in the pre-analytic phase |
| L | p.R3527Q | Present | Present | Absent | Present | Carrier | COUN: Switch of sample of L with C or D in the pre-analytic phase |

*Conclusion on mutation carriership was defined by the end result of the gold reference test in this study, which was the conclusion based on results from analyses in the reference laboratory and the counter-expertise laboratory. ^#^First test of counter-expertise laboratory did not exactly tested for the requested mutation. Subject IDs correspond with those used in text and Figure 1 and Table 2. Switch in the pre-analytic phase meant that erroneous exchange of samples had occurred during the first DNA extraction.. Abbreviations: COUN: counter-expertise laboratory, REF: reference laboratory.

**Supplemental table 3**: Mutation carriership medication use and lipid levels

|  | Cholesterol levels at study visit  (treated and untreated) | | | | | | | | | | LDL-cholesterol at study visit  (untreated only) | | |
| --- | --- | --- | --- | --- | --- | --- | --- | --- | --- | --- | --- | --- | --- |
| Mutation | Carriers* | | | | | Non-carriers* | | | | | Carriers* | Non-carriers* |  |
|  | Treated  yes/all (%) |  |  |  |  | Treated  yes/all (%) |  |  |  |  |  |  |  |
|  |  | TC | HDL | LDL | TG |  | TC | HDL | LDL | TG | LDL | LDL | *p*^#^ |
| All | 153/315 (49%) | 5.9 ± 1.5 | 1.2 ± 0.4 | 4.0 ± 1.3 | 1.4 ± 1.0 | 97/685 (15%) | 4.8 ± 1.0 | 1.3 ± 0.4 | 2.9 ± 0.9 | 1.6 ± 1.0 | 4.6 ± 1.4 | 3.0 ± 0.9 | <0.001 |
| c.313+1/2 | 25/39 (64%) | 6.2 ± 1.8 | 1.2 ± 0.4 | 4.3 ± 1.6 | 1.4 ± 0.9 | 10/101 (10%) | 4.8 ± 1.0 | 1.2 ± 0.4 | 2.8 ± 1.0 | 1.7 ± 0.9 | 5.4 ± 1.8 | 2.9 ± 1.0 | <0.001 |
| p.S306L | 11/23 (48%) | 6.4 ± 1.8 | 1.3 ± 0.3 | 4.5 ± 1.8 | 1.4 ± 1.0 | 7/57 (12%) | 5.0 ± 1.0 | 1.3 ± 0.4 | 3.0 ± 1.0 | 1.5 ± 0.9 | 5.7 ± 1.6 | 3.1 ± 1.0 | <0.001 |
| c.1359-1G>A | 20/26 (77%) | 6.1 ± 2.0 | 1.3 ± 0.4 | 3.8 ± 1.4 | 1.2 ± 0.7 | 13/84 (16%) | 4.8 ± 1.1 | 1.3 ± 0.3 | 2.8 ± 1.0 | 1.4 ± 1.0 | 4.2 ± 0.4 | 3.0 ± 0.9 | 0.008 |
| p.N564H/2393del9bp | 43/86 (50%) | 5.9 ± 1.4 | 1.2 ± 0.4 | 4.1 ± 1.3 | 1.6 ± 1.3 | 39/231 (17%) | 4.8 ± 1.0 | 1.3 ± 0.4 | 2.9 ± 0.9 | 1.5 ± 0.9 | 4.7 ± 1.2 | 3.0 ± 0.9 | <0.001 |
| p.R3527L/Q/W | 50/136 (37%) | 5.6 ± 1.2 | 1.2 ± 0.4 | 3.8 ± 1.2 | 1.3 ± 0.8 | 28/212 (13%) | 4.8 ± 1.0 | 1.2 ± 0.4 | 2.9 ± 0.9 | 1.6 ± 1.1 | 4.3 ± 1.0 | 3.0 ± 0.8 | <0.001 |

*Carriers and non-carriers were defined by the end result of the gold standard test in this study, which was the overall conclusion based on results from analyses in the reference laboratory and the counter-expertise laboratory. ^#^ *p* for difference between the LDL-cholesterol levels between untreated carriers and non-carriers. Abbreviations: TC; total cholesterol, HDL; high-density lipoprotein, LDL; low-density lipoprotein, TG; triglycerides. Treated; treated with cholesterol-lowering medication at the time of lipid assessment. Results for the 8 subjects tested for the *LDLR*-c.313+1/2+c.191-2 mutations were not depicted separately in this table.

**Supplemental file 1: DNA-typing in the counter-expertise laboratory**

PCR-primers were designed for fragments ranging from 75 to 119 bp with Primer 3 v.0.2 (http://frodo.wi.mit.edu) using default settings. Primers with five or more bases at the 3’ end complementary to part of another primer were redesigned to avoid primer-dimers. Amplicon sequences were checked with BLAST for sequence homology in the human genome.

Primers for minisequencing were designed using Assay Design Software Version 1.0.6 (Biotage) and primers with four or more bases at the 3’ end complementary to part of another primer were redesigned to avoid nonspecific primer-extension. Primerlengths were altered by adding a piece of a ‘neutral’ sequence (***Sanchez JJ****, Borsting C, Hallenberg C, Buchard A, Hernandez A, Morling N (2003). Multiplex PCR and minisequencing of SNPs – a model with 35 Y chromosome SNPs. Forensic Science International 137: 74-84*: Sanchez JJ et al., *Forensic Science International* 2003; 137: 74-84). All primer sequences and concentrations are available on request.

Each primer pair was validated in a monoplex PCR containing 2 ng template DNA from a selection of samples (including a reference sample of each mutation), 1 x PCR buffer containing 1.5mM MgCl2 (Applied), 100 µM of each dNTP (GE Healthcare) 0.4 µM of each primer (desalted, Biolegio bv) and 0.6 units of AmpliTaq Gold® DNA polymerase (Applied). In a multiplex PCR, 1 µl template DNA was amplified in a 12.5 µl reaction volume containing 1 x PCR buffer, 4mM total MgCl2, 200 µM of each dNTP and 2.5 units of AmpliTaq Gold® DNA polymerase. Primer concentrations were adjusted (0.06-0.3 µM) to optimize balanced intensity for all markers. All reactions were performed in a GeneAmp 9700 thermal cycler (Applied Biosystems) with a pre-denaturation at 94°C for 10 min followed by 35 cycles of 30 s at 94°C, 30 s at 60°C, 30 s at 72°C and a final extension for 5 min at 72°C. To eliminate excess primers and dNTPs, 2 µl ExoSAP-IT® (USB) was added and incubated at 37°C for 30 min and at 80°C for 15 min.

Minisequencing reactions were performed in a 5 µl reaction volume using 1 µl purified PCR product, 1.5 µl of SNaPshot multiplex Ready Reaction Mix (Applied Biosystems) and 0,4 µM primer (HPLC purified). For multiplex extension primer concentrations were optimized (0.03-0.2 µM). All reactions were performed with a pre-denaturation at 96°C for 2 min, followed by 25 cycles of 10 s at 96°C, 5 s at 50°C and 30 s at 60°C. To eliminate unincorporated ddNTPs 1.25 µl SAP®-reagent (USB) was added and incubated at 37°C for 1 hour and at 75°C for 15 min. 2 µl of the SAP-treated PCR product was analyzed with an ABI3100 Genetic Analyzer using a 36 cm capillary array, polymer POP4 and Genescan 120 LIZ as internal size standard.
